# Prognostic prediction of m6A and ferroptosis-associated lncRNAs in liver hepatocellular carcinoma

**DOI:** 10.1515/jtim-2024-0023

**Published:** 2024-11-06

**Authors:** Yuchen Gao, Jingxiao Li, Mingyue Ma, Wenting Fu, Lin Ma, Yi Sui, Yu Wang

**Affiliations:** Epidemiology and Health Statistics, Shenyang Medical College, Shenyang 110034, Liaoning Province, China; Department of Toxicology, School of Public Heath, Shenyang Medical College, Shenyang 110034, Liaoning Province, China; Integrated Business Department, Shenyang Center for Disease Control and Preventior, Shenyang 110034, Liaoning Province, China; Chengdu Kegene Biotechnology Co., Ltd, Chengdu 610072, Sichuan Province, China; Department of Neurology and Neurosurgery, Shenyang First People’s Hospital, Shenyang Medical College Affiliated Brain Hospital, Shenyang 110041, Liaoning Province, China; School of Public Heath, Shenyang Medical College, Shenyang 110034, Liaoning Province, China

## Introduction

Liver hepatocellular carcinoma (LIHC) is a prevalent malignancy, ranking sixth globally.^[1]^ Current treatments include resection, transplantation, radiotherapy, chemotherapy, and molecular targeted therapy. However, early diagnosis is challenging due to the absence of typical symptoms, leading to poor prognosis and a 5-year survival rate of 30%-40%.^[2]^ Early prediction models and therapeutic targets are urgently needed. N6-methyladenosine (m6A), abundant in messenger RNA (mRNA) and Long non-coding RNA (lncRNA), regulates various ribonucleic acid (RNA) processes and is crucial in cell fate, cycle, differentiation, and circadian rhythm. In LIHC, *METTL3* promotes cell growth, migration, and tumorigenicity, while *METTL14* downregulation indicates poor prognosis.^[3]^ *YTHDF2* facilitates LIHC cell proliferation by recognizing m6A sites. Ferroptosis, an iron-dependent programmed cell death, is more sensitive in cancer cells due to their metabolic activity and reactive oxygen species (ROS) demand. m6A modification is strongly correlated with ferroptosis in LIHC, with roles in *ATG5* expression and autophagy signaling.^[4-5]^ Studies suggest m6A’s pivotal role in regulating ferroptosis and liver cancer treatment. Prognostic models based on lncRNA expression have gained attention, with m6A-associated lncRNA signatures providing new rationale for LIHC diagnosis and therapy.^[6]^ However, additional prognostic markers are needed. This study identified ferroptosis- and m6A-associated lncRNAs, developed an improved prognostic model for LIHC, and investigated the relationship between tumor mutational burden (TMB) and prognosis. Functional enrichment analysis of differentially expressed genes was also conducted.

## Materials and methods

Expression data and clinical information of 424 LIHC patients were obtained from the The Cancer Genome Atlas (TCGA) database. We focused on 22 m6A-related genes and 5 ferroptosis-related genes, and distinguished between mRNA and lncRNA, yielding 16,773 lncRNAs. After filtering, 370 clinical data were matched with expression data. Co-expression analysis was conducted using the “limma” package in R, with a correlation coefficient of 0.4 and *P*-value threshold of 0.001. A prognostic model was developed using m6A/ferroptosis-related lncRNAs, with samples randomly divided into train and test sets. Univariate and lasso-Cox regression analyses identified significant lncRNAs, and a risk score formula was constructed. Survival analysis showed the model’s effectiveness in stratifying high- and low-risk patients. The model was validated through univariate and multivariate Cox regression, with receiver operating characteristic curve (ROC) curves and calibration curves assessing accuracy. Mutation information was analyzed to determine gene mutation frequency, TMB, and tumor immune dysfunction and exclusion (TIDE) scores. Differential expression analysis was performed, followed by Kyoto Encyclopedia of Genes and Genomes (KEGG) and Gene Ontology (GO) enrichment analyses, visualized using various R packages.

## Result

### Screening of m6A and ferroptosis-related lncRNAs and construction of prognostic model

**Figure 1:**
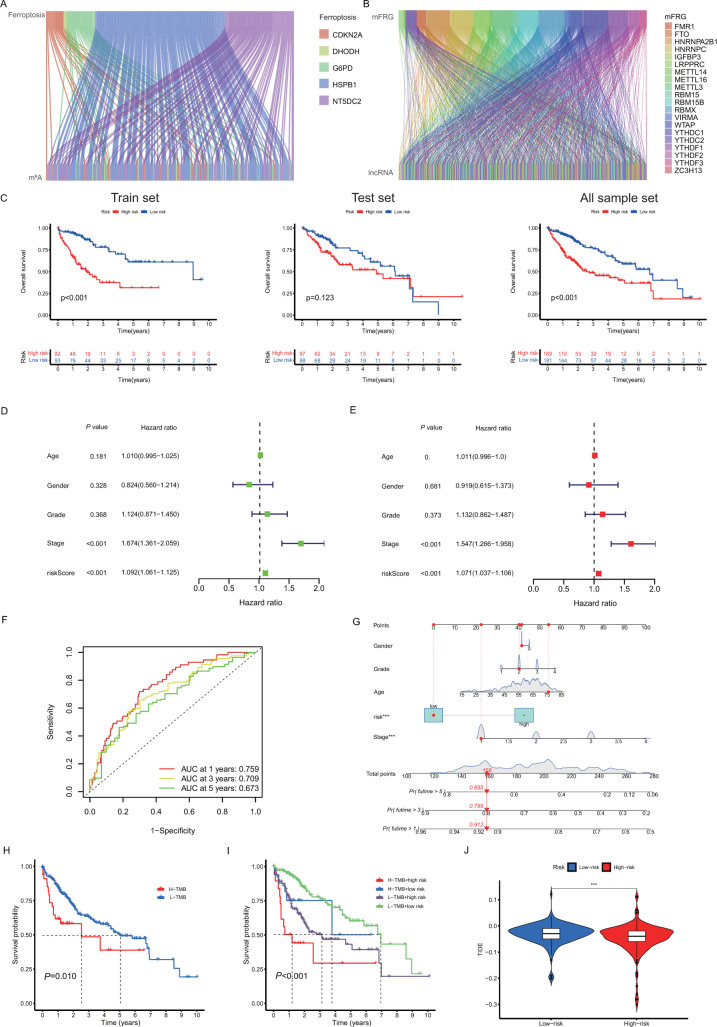
Prognostic prediction of m6A and ferroptosis-associated lncRNAs in liver hepatocellular carcinoma. (A) Co-expression analysis of m6A/ferroptosis genes and lncRNAs. (B) Selection of the optimal penalty parameter for LASSO regression. (C) Prognostic curves in risk groups of the three data sets. (D) Univariate cox regression analysis. (E) Multivariate cox regression analysis. (F) ROC curves for all-sample group. (G) Nomogram for LIHC patient. (H) Survival curves for high-low TMB in all-sample group. (I) Survival curves at change in TMB and risk in all-sample group. (J) Comparison of TIDE in high-low risk groups.

We used 5 ferroptosis-related genes and m6A genes for co-expression analysis, identifying 20 m6A/ferroptosis related genes (mFRGs). From TCGA-LIHC, 16, 773 lncRNAs were analyzed, revealing 593 m6A/ferroptosis related lncRNA (mFlncRNAs). Univariate cox analysis screened 80 mFlncRNAs, and lasso regression further reduced to 13, ultimately yielding 7 for the risk model. High-risk included *ELFN_1_-AS1*, *AL603839.2*, *etc*., while low-risk were *AC073573.1*, *AC069307.1* (Figure 1A-B) (Supplemental Figure 1A-B) (Supplemental Table 1).

### Prognostic impact of mFlncRNAs at different risks on LIHC

Risk scores classified samples into high- and low-risk groups. In train, test, and all-sample sets, low-risk patients had lower fatality and higher survival rates than high-risk patients (Figures 1C). Risk heatmaps showed that high-risk mFlncRNAs (ELFN_1_-AS1, *etc*.) increased, while low-risk mFlncRNAs (AC073573.1, *etc*.) decreased from low- to high-risk groups (Supplemental Figure 1C-K), consistent with prior univariate cox analysis.

In all clinical subgroups (age, sex, tumor differentiation, clinical stage), low-risk patients had better survival than high-risk patients, confirming the model’s predictive power (Supplemental Figure 2). Principal component analysis (PCA) showed that the seven mFlncRNAs in the model effectively distinguished high- from low-risk patients, whereas other groups (TCGA-LIHC genes, mFR Genes, all mFlncRNAs) did not (Supplemental Figure 3A-D).

### Independent prognostic analysis and model evaluation

Univariate and multivariate analyses showed risk score and tumor stage as significant high-risk factors. The risk-prognostic model predicted LIHC prognosis with high accuracy, with ROC curves area under curve (AUCs) of 0.759, 0.709, and 0.673 at 1, 3, and 5 years. A nomogram model, including risk score, age, gender, grade, and stage, predicted 1-, 3-, and 5-year survival rates accurately (Figure 1D-G). Calibration curves showed excellent agreement between actual and predicted observations (Supplemental Figure 3E-F). The constructed model had the highest c-index, indicating its superior accuracy in predicting patient survival (Figure 1D-G).

### Genetic mutation associated risk score

Our analysis showed *TP53* mutations were more frequent in high-risk LIHC patients (34%) than in low-risk (18%), while *CTNNB1* mutations were more common in low-risk (34%) than high-risk (18%) patients (Supplemental Figure 3G-H). TMB stratified patient prognosis, with lower levels associated with better outcomes. Combining TMB with risk classification predicted survival, with low TMB and low risk patients having the best prognosis. High-risk patients had significantly different TIDE scores, with higher scores in the low-risk group indicating greater immune escape potential and poorer immunotherapy outcomes (Figure 1H-J).

### Differential gene expression and functional enrichment analysis

Analysis of gene expression differences between high- and low-risk groups identified 691 genes with differential expression, with 594 genes upregulated and 97 genes downregulated in the high-risk group. KEGG analysis showed these genes were enriched in cell cycle, cellular senescence, and oocyte meiosis, suggesting association with cell growth. GO analysis revealed enrichment in nuclear division, organelle fission, chromosome segregation (biological processes), antigen binding, tubulin binding (cellular components), and chromosomal region, spindle (molecular function), indicating diverse functional roles (Supplemental Figure 3I-K).

## Discussion

LIHC is a complex disease caused by multiple factors, including chronic hepatitis B virus (HBV), hepatitis C virus (HCV), aflatoxin-contaminated food, heavy alcohol consumption, obesity, smoking, and type 2 diabetes.^[7]^ Current early diagnosis relies on alPhafetoProtein (AFP), but its accuracy is limited.^[8]^ Thus, exploring additional molecules in LIHC’s molecular mechanism is crucial for better disease understanding and management. Studies have shown alterations in lncRNA levels are implicated in various cancers and could serve as diagnostic markers and therapeutic targets.^[9]^ Our study presents a novel risk-prognostic model for LIHC patients, focusing on lncRNAs, m6A modification, and ferroptosis. We identified *ELFN_1_-AS1*, *AC018690.1*, *AL603839.2*, *LINC02313*, and *AC004801.6* as high-risk factors, while *AC069307.1* and *AC073573.1* were low-risk factors. Our model, validated by PCA and ROC curve, showed higher predictive power than conventional clinical models. Additionally, we found that high TMB was associated with poor prognosis in LIHC patients, but our risk prognosis model demonstrated stronger predictive potential. KEGG and GO analysis identified gene enrichment in cell cycle regulation, bile secretion pathways, and xenobiotics metabolism. While our study identified mFlncRNAs’ potential in early LIHC diagnosis, further validation is needed. Leveraging mFlncRNAs can devise more rational therapeutic strategies, ultimately enhancing LIHC patients’ survival rates.

## Supplementary Material

Supplementary Material Details
