# Supplementary material for: Prognostic prediction of m6A and ferroptosis-associated lncRNAs in liver hepatocellular carcinoma
Source: J Transl Int Med. 2024 Nov 6;12(5):526–9. doi: 10.1515/jtim-2024-0023 (PMC11538885; doi:10.1515/jtim-2024-0023)
Supplement: Supplementary file 1 — Supplementary Material Details [file jtim-2024-0023_sm.zip › jtim-2024-0023_sm/6 JTIM-D-24-00292 SI.pdf]

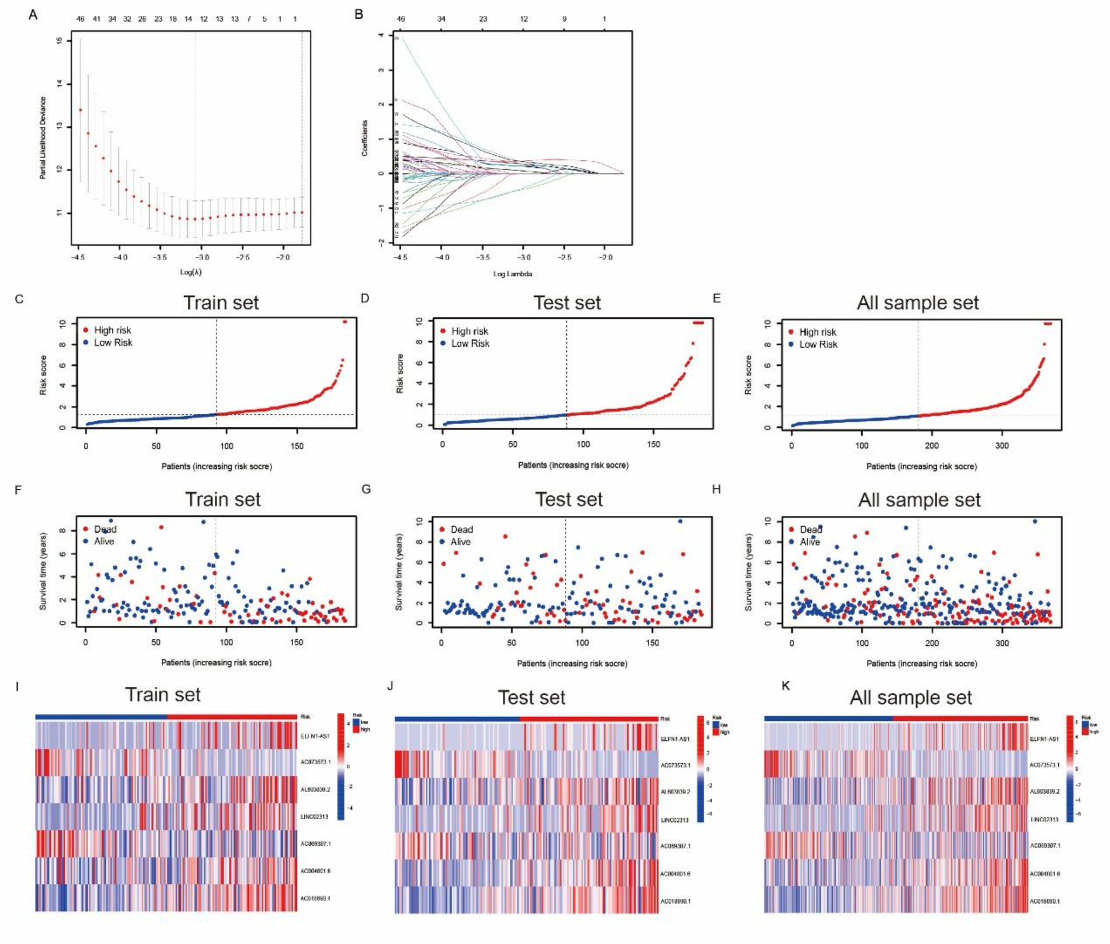

Supplementary Figure 1. Survival analysis of prognostic models. (A) Selection of the optimal penalty parameter for LASSO regression. (B) LASSO regression analysis. **(**C-E) Risk curve for patients respectively, in the three data sets. (F-H) Survival status map for patients respectively, in the three data sets. (I-K) Risk heatmap for patients respectively, in the three data sets.

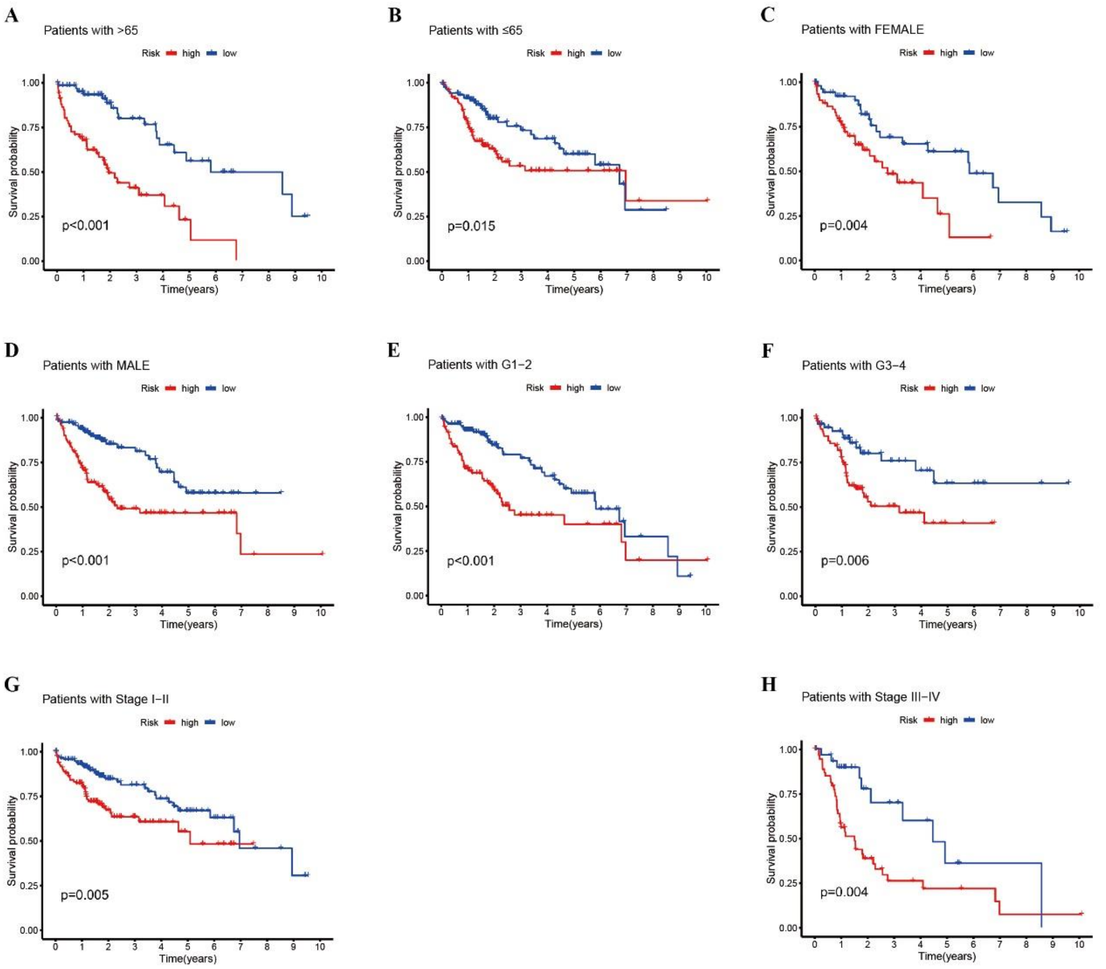

Supplementary Figure 2. Model validation for clinical subgroups. (A-B) Grouped by age. (C-D) Grouped by gender. (E-F) Grouped by tumor grade. (G-H) Grouped by tumor stage.

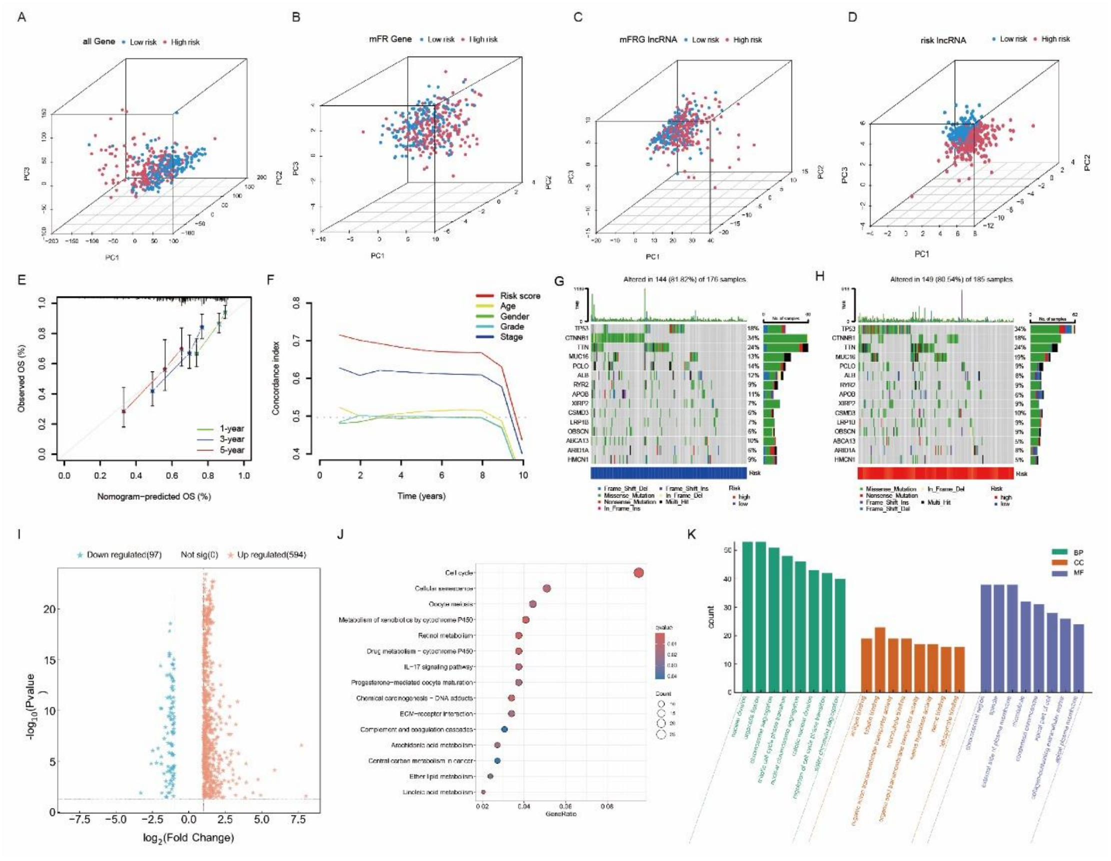

Supplementary Figure 3. Principal Component Analysis, Clinical Features, and Mutations in High- and Low-Risk LIHC Patient Groups. (A-D) Principal Component Analysis. (B) Multivariate cox regression analysis. (E) Calibration curves for nomogram. (F) C-index analysis of model and clinical traits. (G) Waterfall plot of mutations in samples from the low-risk group. (H) Waterfall plot of mutations in samples from the high-risk group. (I) Volcano plot of differentially expressed genes in high-low risk groups. (J) KEGG analysis of differentially expressed genes. (K) GO analysis of differentially expressed genes.

Supplementary Table 1. mFlncRNAs significantly associated with LIHC prognosis.

mFlncRNAs significantly associated with LIHC prognosis.				
id	HR	HR.95L	HR.95H	pvalue
AC010761.1	2.106951886	1.055320716	4.206537581	0.034634829
ELFN1-AS1	1.460308047	1.164348664	1.831495718	0.001050114
NRAV	2.937531925	1.789125295	4.823079654	2.05E-05
AC012467.2	2.370739701	1.332025277	4.2194445	0.003338964
AL357079.3	2.244292962	1.067690039	4.717521671	0.032944007
CTC-338M12.4	4.385927752	1.322036032	14.55055822	0.015682082
AC006252.1	5.836611683	1.571275197	21.68050257	0.008416401
MAPKAPK5-AS1	1.658242047	1.089966209	2.522799939	0.018159642
LINC00665	1.426048462	1.026604502	1.980913011	0.034298001
AC005332.5	1.869039114	1.144698447	3.051727046	0.012412085
AC073573.1	0.192766835	0.037278038	0.996808159	0.0495559
AL161729.4	2.759426338	1.248872263	6.097047665	0.012093586
SNHG1	1.401601069	1.039189751	1.890401205	0.02698029
AC012073.1	1.949968068	1.015125348	3.745720146	0.044959178
AL133215.2	2.876970156	1.246179463	6.641866221	0.013303741
SNHG26	1.943039785	1.065764354	3.542437491	0.030171851
AC016405.3	1.447163771	1.064832227	1.966772724	0.018211899
LINC02362	0.718189604	0.529076564	0.974899179	0.033753634
AC125437.1	2.289569937	1.132443537	4.62904359	0.021097044
MCM3AP-AS1	23.35878761	4.010060661	136.0660112	0.000457192
AC004839.2	5.452572039	1.191227269	24.95790906	0.028857073
SNHG20	2.233765556	1.121553942	4.448924275	0.022236653
DDX11-AS1	6.689580944	1.277297008	35.03530732	0.02447002
NIPBL-DT	2.401706207	1.308327306	4.408830021	0.004696807
AC022211.3	4.790840011	1.503120781	15.26966316	0.008071674
AL355488.1	1.574553971	1.054029983	2.352134427	0.026627627
RNF216P1	1.840221056	1.066423161	3.175487609	0.028452044
AC004812.2	2.347399431	1.123129042	4.906189655	0.023287296
AC068756.1	1.948939487	1.257686139	3.020121641	0.002827534
AC090181.2	2.458630972	1.122731468	5.384071286	0.024485215
AC016065.1	3.140995376	1.488302319	6.628930044	0.00266974
AL358472.4	2.052545284	1.038038735	4.058559666	0.03870652
AL050341.2	1.565870262	1.07073426	2.289970322	0.020756648
AL137785.1	1.503593448	1.020313938	2.215781998	0.039244243
AC027097.1	2.583384186	1.076862026	6.197519916	0.033517707
AC004943.2	4.945884404	1.703592406	14.35893495	0.003285991
AL603839.2	3.610835345	1.464582535	8.902285516	0.005291312
AC091057.1	2.45636647	1.105720637	5.456836051	0.027332412
AL117336.2	2.207281581	1.3915218	3.501268884	0.000769442
AL031985.3	2.906993017	1.641581952	5.147844366	0.000252257
AC026412.3	9.155916811	2.363855001	35.46360187	0.001349816
SNHG4	1.616884588	1.137057043	2.299194916	0.007472268
LINC02313	1.792957659	1.059805175	3.033290688	0.029518737
AC092910.3	3.020242241	1.246613502	7.317314614	0.014357508
AP001469.3	2.377529683	1.1890937	4.753744298	0.014290352
AC069307.1	0.458427286	0.211661825	0.992883701	0.047920079

AC083862.2	1.518735574	1.049600041	2.197558739	0.026639109
AC012676.3	4.061830985	1.300512432	12.6861309	0.015858245
WAC-AS1	1.847893275	1.209630705	2.822935579	0.004507613
AC026356.1	2.873485629	1.243399036	6.640603231	0.013523469
TRAF3IP2-AS1	8.294688811	1.760421003	39.08261851	0.0074713
SCAT2	1.785534563	1.107941095	2.877529943	0.017267818
AC145207.5	2.66088453	1.316005505	5.380149592	0.006441812
NRSN2-AS1	2.293637112	1.014060382	5.187828351	0.046207447
LINC02820	1.797005743	1.167888384	2.765015634	0.007679523
AC006504.7	1.675538802	1.071616474	2.619808808	0.023619106
CAPN10-DT	2.277586137	1.054833291	4.917742599	0.036091784
AC089999.2	2.693808349	1.083531013	6.697181099	0.032956336
AL606489.1	1.56196297	1.06224564	2.296764728	0.023394572
C2orf27A	4.068811014	1.973294594	8.389635848	0.000144164
AC009271.1	2.645706282	1.316513144	5.316894679	0.006291819
LINC01224	2.662106616	1.48604901	4.768894962	0.00099593
TMCC1-AS1	3.654630301	1.407003839	9.492740718	0.007788651
AC098484.4	4.10946584	1.206769395	13.99414797	0.023784751
AL355574.1	2.377945762	1.23354673	4.58403878	0.009688719
AC020915.1	3.179344657	1.555617652	6.497890041	0.001516205
TMCO1-AS1	2.909044694	1.039745534	8.139050143	0.04193007
ZNF674-AS1	2.115234208	1.015411353	4.406308577	0.045412933
TMEM147-AS1	2.125673189	1.150680852	3.926793863	0.016032083
LINC00205	1.827791783	1.043225541	3.202397441	0.035042825
AL357079.1	4.213769505	1.692432711	10.49132017	0.001998169
BACE1-AS	2.641899666	1.134015043	6.154798286	0.024358975
NIFK-AS1	2.436181918	1.180459106	5.027689911	0.016006868
AC004801.6	3.838484043	1.190251096	12.37886678	0.0243543
AC025176.1	2.519837659	1.309706502	4.848095217	0.005639287
KDM4A-AS1	4.042690134	1.258834324	12.98291857	0.018943012
AC018690.1	4.552213532	1.995992074	10.38212942	0.000314604
FOXD2-AS1	1.534246693	1.087491249	2.164535042	0.014784791
ZNF337-AS1	3.096049712	1.060098584	9.042106046	0.038763196
SNHG10	2.386668648	1.017607873	5.597624969	0.045489874
